# Individual and community level factors of safe child feces disposal among youngest children in East Africa: a multilevel modeling analysis using recent national demography and health survey data

**DOI:** 10.1186/s13690-024-01335-z

**Published:** 2024-07-18

**Authors:** Bewuketu Terefe, Nega Tezera Assimamaw, Bogale Chekole

**Affiliations:** 1 Department of Community Health Nursing, School of Nursing, College of Medicine and Health Sciences, University of Gondar, Gondar, Ethiopia; 2 Department of Pediatric and Child Health, School of Nursing, College of Medicine and Health Sciences, University of Gondar, Gondar, Ethiopia; 3 Department of Comprehensive Nursing, College of Medicine and Health Sciences, Wolkite University, Southern, Ethiopia

**Keywords:** Factors, Child feces disposal, Youngest children, East Africa, Multilevel modeling

## Abstract

**Background:**

The Joint Monitoring Program (JMP) for water supply and sanitation developed by the WHO and UNICEF defines safe child feces disposal practices as either burial or defecation into a toilet. Children become exposed to fecal-oral illnesses when their stools are not disposed of appropriately, and this vulnerability persists until all children’s stools are properly disposed of. Data on the elements influencing child feces disposal in East Africa is scarce. Hence, this study aimed to assess the prevalence and associated factors of safe child feces disposal in East Africa.

**Methods:**

Data from the Demographic and Health Surveys, which were collected between 2015 and 2022 in 10 East African nations, were used in this analysis. For a weighted 44,821 children under the age of two, we examined additional features as well as how child feces were disposed of. Both bivariable and multivariable multilevel logistic regression were carried out to choose potential components and identify important explanatory variables connected to the outcome variable. With 95% confidence intervals, adjusted odd ratios (AORs) were used to present the results. *P* values of ≤ 0.2 and < 0.05 were used to investigate significant factors in the binary and multivariable multilevel logistic regression models respectively.

**Results:**

Approximately 65.54% (95% CI: 65.10, 65.98) of children’s waste was disposed of properly. Women age from 35 to 49 years (AOR = 1.12, 95% CI: 1.05–1.19) 15–24 years old, primary (AOR = 1.62, 95% CI, 1.53,1.72), and secondary/higher education (AOR = 1.22, 95% CI, 1.14,1.31), women from highly educated community (AOR = 1.33, 95% CI, 1.22,1.46), employed (AOR = 1.29, 95% CI, 1.24,1.35), poorer(AOR = 1.51,95% CI, 1.42,1.61), middle(AOR = 1.67, 95% CI, 1.56,1.78), richer(AOR = 1.96,95% CI, 1.82,2.11), and richest(AOR = 2.08, 95% CI, 1.91,2.27), mass media exposure (AOR = 1.37,95% CI,1.31,1.44), community level mass media exposure (AOR = 1.23, 95% CI, 1.34,1.34), had ANC visit(AOR = 1.71, 95% CI, 1.55,1.88), modern contraceptive(AOR = 1.17, 95% CI, 1.12,1.23), health institution delivery (AOR = 2.22, 95% CI, 2.09,2.34), had an improved toilet facility (AOR = 1.12, 95% CI, 1.07,1.17), children who’s their age group from 6 to 11 months old, (AOR = 2.12, 95% CI, 2.01,2.25) and 12–23 months old (AOR = 3.10,95% CI, 2.94,3.27) were the factors associated with higher odds of safe child feces disposal as compared to less than six months old children respectively. Finally, women from high community poverty level (AOR = 0.87, 95% CI, 0.79,0.95), and rural women were the factors associated with lower odds of safe child feces disposal (AOR = 0.91, 95% CI 0.85–0.98) compared to their counterparts respectively.

**Conclusions:**

East Africa has a Slightly lower proportion of properly disposing of child feces. There was a strong correlation between characteristics such as residence, mother’s age, education level, work status, place of delivery, ANC visit, child’s age, wealth index, media exposure, and poverty. Acting on these factors and strengthening and using links between mother and child health care is, thus, strongly advocated.

**Table:** 

Text box 1. Contribution to the literatures	
Examining child feces by highlighting the health hazards connected to improper disposal, and the requirement for awareness and education, correct disposal adds to the body of knowledge already available. There is no current data to support the claim that East Africa has a poor rate of properly disposing of child feces. For child’s, care givers, and other community members, the improper disposal of child feces may pose a greater threat to their health than that posed by the excrement of adults. Interventions to enhance child feces disposal could help avoid diarrhea and helminth infections that are spread through the soil. One of the most important means of raising awareness among people, especially in developing nations, is education, and women empowerment. This study lends credence to such notion.	

## Introduction

The World Health Organization (WHO), and UNICEF created a safe sanitation system to separate human waste from human activity at all points in the sanitation chain, including safe toilets, containment (in some in-situ treatment systems), transportation (in sewers or by emptying and transport), care, and eventual disposal or final usage [1, 2]. Access to proper sanitation and hygiene for all facilities is still a challenge and a global issue when it comes to ending open defecation [3]. Although open defecation is a frequent behavior among children in many low-income nations, the issue has received little attention in the area thus far [4, 5].

With a focus on the needs of women, girls, and those who are vulnerable, the Sustainable Development Goal (SDG 6.2) indicator 6 aspires to “achieve access to adequate and equitable sanitation and hygiene for all and end open defecation by 2030“ [6, 7]. By 2030, current rates of advancement in securely supervised drinking water administrations, sanitation administrations, and basic hygiene services must be quadrupled, particularly in least developed nations [7]. One of the top priorities for reducing worldwide inequities in water, sanitation, and hygiene (WASH) is the elimination of open defecation [8, 9].

The incidence of diarrheal illnesses increased by 23% [10], when child feces were not disposed of safely. Additionally, it has an impact on children growth [11, 12]. On the other hand, helminth infections were reduced by 35% as a result of safe child feces disposal [4, 13]. Additionally, numerous studies revealed that bettering water availability and hygiene standards, especially the proper disposal of children’s waste, might avert 361,000 deaths of children under the age of five every year [14, 15].

According to numerous studies, hazardous child soiled disposal and open defecation are two of the main causes of enteric infections, including diarrheal disorders, and other contagious infections as a result [16–18]. Additionally, it quickens the spread of gastrointestinal illnesses [13, 19]. Due to the fact that children’s feces are regarded to constitute a bigger public health danger than those of adults (children’s feces contain higher concentrations of pathogens), this problem is more severe in children. Parents also believe that infants’ feces are safe, hence they frequently permit open defecation in the home [20, 21].

Previous public health literatures showed that wealth [22–24], media exposure [22–25], types of residences [22], latrine facility [23, 25–27], mothers’ age [24, 26, 28], source of drinking water [22, 27], child’s age [24, 27], education level of mothers/care givers [23, 25, 29], number of children 5 years of age in the household [23, 29], and occupational status of mothers [28], were all linked to the disposal of child feces. Although the scope of safe child feces disposal is understood, the potential influences on it have not yet been well investigated in East Africa. There is very little data available on the East African regional pooled estimate and associated determinants of safe child feces disposal as compared to other regions [30–33]. Therefore, the current study’s objective is to evaluate, using the East African combined most recent DHS, variables that are significantly related to safe child feces disposal practices in the region.

## Methods

### Study setting, period, and time frame

The data came from the most recent standard Demographic and Health Survey (DHS) data set of East African countries between 2015/16 and 2022 (Table 1). We used a standardized data set [34] to obtain all parameters and a large sample size that is representative of the population source. DHS collects data that is cross-nationally comparable. The surveys are population-based and nationally representative of each country, with large sample sizes [34]. Eastern Africa is made up of 14 countries located in the Great Lakes region, the Horn of Africa, or the Indian Ocean islands. These countries confront similar economic, social, and environmental issues, and they are concerned about not meeting all of the Sustainable Development Goals targets [35]. East Africa is the region of the African continent that falls inside the Saharan desert in the horn and Eastern Africa. According to United Nations estimates, they cover an area of 6,667,493 Km2 (2,574,332 square miles) and house 6.03% of the world’s population, with a total population of 486,766,759 persons.

**Table 1:** Countries, sample size, and survey year of Demographic and Health Surveys included in the analysis for 10 east African countries

Country	Survey year	Weighted sample	Weighted frequency
Burundi	2016/17	5,173	11.54
Ethiopia	2016	4,069	9.08
Kenya	2022	6,398	14.28
Madagascar	2021	4,577	10.21
Malawi	2015/16	6,271	13.99
Rwanda	2019/20	3,121	6.93
Tanzania	2015/16	3,917	8.74
Uganda	2016	5,388	12.02
Zambia	2018	3,649	8.14
Zimbabwe	2015	2,271	5.07

### Sample size determination and sampling technique

For roughly 12 of the 14 East African nations, demographic and health survey Reports were available. All of the surveys carried out in the countries listed used the most current conventional census frame. DHS samples are usually divided between urban and rural areas within each administrative geographic region. Enumeration areas (EAs) within each stratum were picked in the initial sampling with a probability proportionate to their size. In the second stage of sampling, the systematic sampling approach selects a predetermined number of households in designated EAs. Following the listing of the households, an equal probability is used to select a fixed number of households in the defined cluster. DHS standards established four sampling weighting methods, one of which we employed for women (v005). Individual sample weights are calculated by dividing (v005) by 1,000,000 and then used to estimate the number of cases [34].

### Data sources, and data quality control

The most recent attached demographic and health surveys (KR data sets), which were carried out in ten East African nations between 2015 and 2022, were the source of the secondary data used in this study. Data on fundamental health indicators like morbidity, mortality, fertility, maternal health, and child health are gathered by the DHS, a nationally representative survey. The study participants were chosen by the DHS using a two-step stratified sampling approach. Data collection for the DHS surveys used pretested and typical DHS questionnaires. Data were gathered by skilled data collectors who conceived the questionnaire for the setting of the various nations. The datasets for every country in the East African region may be found at https://dhsprogram.com/data/dataset_admin/index.cfm. The investigation was limited to the nations with information on feces disposal among children under two. To deal with missing data, we eliminated the cases that were not complete from the analysis. To generate a single data collection that represents the East African countries, a code was assigned to each nation, which was then added together. A weighted sample of 44,821 under-two-year-old in total was used in this investigation (Table 1).

### Variables of the study

#### Outcome variable

The outcome variable of this study was the numbers of youngest children under age 2 living with mother whose last fecal matter is disposed of appropriately: if the child used a toilet or latrine, if the fecal matter was put or rinsed into a toilet or latrine or if it was buried. Regarding the coverage: the population base was all youngest child under age 2 living with the mother (KR file), and the time period was estimated by the disposal of child’s most recent fecal matter. To select the cases for the denominator, first filter or keep only children living with the mother born in the preceding 24 months (keep/select if b19 < 24 & b9 = 0), then keep only the youngest child (keep/select if the first entry in the dataset (_n = 0) or the first for this respondent (caseid ≠ caseid [_n_1]) as the youngest child is the first listed for the respondent in the data file) was done using Stata 17. Then the outcome variable was recategorized as Yes = “1” if the mothers’ ways of disposal of their child feces is appropriate, and No= “0”, if it was not followed the appropriate ways. This classification, and the analysis has been made according to the guide to DHS statistics book [36].

#### Independent variables

Various sociodemographic, maternal reproductive health, and child-related factors were included in the study. Sociodemographic-related factors encompassed maternal age, educational status, types of places of residence, marital status, household wealth index, current employment status, mass media exposure, age of the mother at first birth, total children born, under five children, marital status, family size, sex of the household head, types of toilet facility, types of water sources, community-level education, community-level poverty, and community-level mass media. Maternal and reproductive health-related factors comprised ANC follow-up, place of delivery, PNC checkup, and modern contraceptive utilization. Additionally, child-related factors consisted of age of the child, sex of the child, size at birth, twin status, and birth order.

### Operational definitions for some factors

Due to the unavailability of observed or recorded aggregated data at the community level during the survey, all community-level components had to be estimated using aggregate values derived from individual records. Each component was estimated based on the value of its corresponding distinct variable, even if the methodology used was consistent with that of other relevant literature. In this study, a cluster or primary sample unit in the dataset, representing a group of families, was referred to as a community level factor. These components were then combined at the individual, group, and community levels to generate variables for analysis. The community variables considered in this study included community women’s education, which represented the percentage of women with primary or post-primary education in the community. Community media exposure was another variable, indicating the level of media exposure within the community. Additionally, community poverty was assessed by calculating the percentage of households classified as impoverished. Apart from these variables, other readily available community factors, such as place of residence, were also taken into account during the analysis. The continuous community-level variables were further split into low and high categories using the mean/median value based on their distribution in order to make the results easier to grasp [37–39].

#### Community women’s education

The educational achievement of women in the given community can be assessed by examining the distribution of women’s educational attainment. The median serves as a benchmark in determining the level of educational achievement. If the proportion of women in the community who have attained at least a secondary education falls below the median value of 72%, it is considered to be a low proportion, indicating a lower level of educational attainment among women. Conversely, if the proportion exceeds the median range of 73–100%, it is considered to be a high proportion, indicating a relatively higher level of educational achievement among women in the community.

#### Community level women media exposure

The media exposure variable in this study was determined by individual responses regarding their exposure to various media outlets such as radio, books/newspapers, or television. To assess the level of media exposure, a range was defined based on the proportion of women in the community who reported being exposed to media. A low level of media exposure was considered when the proportion of women exposed to media fell between 0% and 44%. Alternatively, a high level of media exposure was defined when the proportion of women exposed to media ranged between 45% and 100%. This categorization allowed for a comprehensive analysis of the varying degrees of media exposure within the community.

#### Community poverty

The variable based on the wealth index of each household was determined using a similar process. To ascertain the level of the variable, households were categorized into wealth quintiles. In the two lowest quintiles of wealth within the community, the variable was considered high if between 49% and 100% of women were present in the household. Conversely, it was considered low if between 0% and 48% of women were present in the household. This approach allowed for the assessment of the variable in relation to the wealth distribution within the community, providing insights into the presence of women across different socioeconomic strata.

#### Improved/unimproved toilet facility

The identified types of toilet facilities were categorized as follows: flush-to-piped sewer system, flush-to-septic tank, flush-to-pit latrine, flush-don’t know where, pit latrine-ventilated improved pit (VIP), pit latrine-with slab, and composting toilet, representing improved toilet facility types. On the other hand, unimproved toilet facility types included flush-to-somewhere else, pit latrine-without slab/open pit, bucket toilet, hanging toilet/latrine, and others.

### Data processing, and analysis

The data used in this investigation were taken from the most recent regular Demographic and Health Survey (DHS) after permission was obtained through an online request detailing the objectives of the study. The regular DHS data set was utilized to gather all parameters and a sizable sample size representative of the source [36]. The DHS files for child’s records (KR) were downloaded in STATA format. Following access to the data, it was cleaned, coded, and merged to provide suitable variables for the analysis. The data were then weighted using sample weight for probability sampling and non-response to restore representativeness before any statistical analysis. Finally, a total weighted sample of 43,150 children aged 0 to 23 months from ten East African countries were included in this study. Software such as STATA 17 and Microsoft Excel 2019 were utilized to give both descriptive and analytical statistics to define variables in the study utilizing statistical measurements.

The representativeness of the survey results was therefore guaranteed. For the multivariable analysis, variables with a *p*-value of 0.2 in the bivariable analysis were considered. The multivariable logistic model’s Adjusted Odds Ratio (AOR) with 95% CI was given to identify the contributing factors of child feces disposal. Descriptive studies like frequency count and proportion were utilized to summarize the descriptive data for categorical data. Bivariable multilevel logistic regression was used to find possible variables for multiple multilevel logistic regression. To account for the hierarchical nature of the data, a two-level multilevel fixed effect binary and multiple logistic regression analysis were employed to evaluate the effect of explanatory variables on the safe child feces disposal among children in East Africa. The data is divided into two levels, with a group of J EAs and within-group j (j = 1, 2…J), and a random sample nj of level-one units (households). The response variable is represented as; Y_ij_ = 1 if the i^th^ households was in the j^th^ EAs had an exposure of safe child feces disposal, 0 if i^th^ households was in the j^th^ EAs had no exposure of child feces disposal.

To account for the nested effect, acceptable deductions and conclusions from this data require adequate modeling techniques such as multilevel modeling, which includes variables assessed at multiple levels of the hierarchy [40]. Four models were fitted to the data. The initial model used to calculate the extent of cluster variation in child feces disposal was an empty model with no explanatory parameters. To compute differences between clusters (EAs), the Intra-Class Correlation coefficient (ICC), proportional change in variance (PCV), and median odds ratio (MOR) were employed. The fraction of variance explained by the population grouping structure is represented by the ICC. Unlike the null model, PCV assesses the total variation attributable to individual and community level components in the multilevel model [41].

When two clusters (EAs) are picked at random, the MOR is defined as the median value of the odds ratio between the clusters at high and low risk of child feces disposal. The second model included just community-level variables, the third included only individual-level variables, and the fourth included both individual and community-level variables. The model with the lowest deviance (-2LLR) was chosen as the best-fitted model for the data. Variables with a *p*-value of < 0.2 in the bivariable analysis were considered for the multivariable analysis. In the multivariable multilevel binary logistic model, the best-fitted model’s Adjusted Odds Ratio (AOR) with 95% CI was reported to determine the associated factors of child feces disposal among under two years old children. The statistical significance of the final model was set at p less than 0.05.

## Results

### Sociodemographic characteristics of the study participant

In this study, a weighted total of 45,144 under-two-year-old children were enrolled in East African countries. In total, 65.54% of children under the age of two had their feces appropriately disposed of.

About nearly half (19,842 (44.27%) of the study women were found in the age group of 25–34 years of reproductive age. Regarding marital status, the majority of mothers, 30,537 (68.13%), were married. With respect to place of residence types 35,368 (78.91%), educational status 22,261 (49.67%), wealth index 10,660 (23.78%), place of delivery 34,709 (77.44%), and ANC follow-up 42,131 (94.00%) of mothers were from rural areas, primary educational status, poorest household’s wealth index, institutional delivery, and had at least one ANC follow-up during their pregnancies, respectively. Furthermore, more than half, 25,532 (56.96%) of women had given birth before reaching the age of twenty, and 30,349 (67.82%) of them had no history of postnatal care follow-up. However, only less than half, 19,724 (44.01%), of them have utilized modern contraception. Similarly, about 25,222 (56.27%) women had no exposure to at least one type of mass media (either listening to radio, watching television, or reading magazines or newspapers); however, 27,679 (61.76%) and 38,380 (64.87%) of them were employed. About two-thirds (32,201, or 71.85) of women have an improved source of drinking water; however, less than half (21,417, or 47.78%) of them have an improved toilet facility type.

In addition to this, regarding child-related characteristics, the majority of mothers, 26,676 (59.52%), have more than one child under five, and almost all of them were single, 44,219 (98.66%). Regarding order of birth, about 17,150 (38.26%) were in the 4th or above birth order, the average weight was 22,388 (53.61%), and about 22,532 (50.27%) of the children were male (Table 2).

**Table 2:** Sociodemographic, maternal reproductive health, and child related characteristics on disposal of youngest children’s stools among under two years old in east African countries recent DHS (weighted *n* = 44,821, and unweighted *n* = 45,144)

Dependent variables	Frequency (weighted)	Percentage
**Maternal age**		
15–24	16,701	37.26
25–34	19,842	44.27
35–49	8,279	18.47
**Maternal education**		
Not educated	8,953	19.97
Primary	22,261	49.67
Secondary & higher	13,608	30.36
**Maternal employment**		
No	17,142	38.24
Yes	27,679	61.76
**Wealth index**		
Poorest	10,660	23.78
Poorer	9,490	21.17
Middle	8,665	19.33
Richer	8,385	18.71
Richest	7,622	17.00
**Mass media exposure**		
No	25,222	56.27
Yes	19,599	43.73
**Postnatal checkup**		
No	30,349	67.82
Yes	14,402	32.18
**Modern contraceptive utilization**		
No	25,097	55.99
Yes	19,724	44.01
**Age at first birth**		
< 20	25,532	56.96
>=20	19,289	43.04
**Family size**		
<=5	21,702	48.42
> 5	23,119	51.58
**Total children born**		
> 3	20,250	45.18
3–5	16,942	37.80
> 5	7,629	17.02
**Marital status**		
Unmarried	14,284	31.87
Married	30,537	68.13
**Place of delivery**		
Home	10,111	22.56
Health facility	34,709	77.44
**ANC follow-ups**		
No	2,690	6.00
Yes	42,131	94.00
**Twin status**		
Single	44,219	98.66
Multiple	602	1.34
**Birth order**		
1st	11,045	24.64
2nd or 3rd	16,625	37.09
4th and above	17,150	38.26
**Child size**		
Large	11,949	28.67
Average	22,388	53.71
Small	7,347	17.63
**Sex of the child**		
Male	22,532	50.27
Female	22,289	49.93
**Number of under five children**		
No or one only	18,145	40.48
More than one	26,676	59.52
**Source of drinking water**		
Unimproved	12,620	28.16
Improved	32,201	71.84
**Types of toilet facility**		
Unimproved	23,403	52.22
Improved	21,417	47.78
**Sex of the household head**		
Male	|35,116	78.35
Female	9,705	21.65
**Residence type**		
Urban	9,452	21.09
Rural	35,368	78.91
**Community mass media**		
High	22,542	50.29
Low	22,279	49.71
**Community education**		
High	22,817	50.91
Low	22,004	49.09
**Community poverty**		
High	22,970	51.25
Low	21,851	48.75

### Factors associated with disposal of youngest child’s stools in East Africa

#### Random effect analysis

The random-effects model discovered significant clustering of appropriate child feces disposal across communities (CLV, or community level variance = 0.35). The null model’s ICC score revealed that cluster variability accounted for 10.00% of the overall variation regarding appropriate child feces disposal. The ICC values of model I, model II, and model IV were observed at 9.34%, 8.22% and 8.88% respectively. The 1.76 MOR value of the null model also implies that there is variation in the practice of childhood waste disposal among clusters. That is, if we randomly select households from different clusters, those in the cluster with the highest households’ safe or appropriated child feces disposal had 1.76 times greater odds of practicing appropriate waste disposal than their counterparts. As demonstrated in Table 3, the PCV increases from the null model to model III, suggesting that model III best captures the variety of appropriate waste disposal. Model III also has the lowest deviation and AIC measures. As a result, it was picked as the model with the best fit (Table 3).

**Table 3:** Individual and community level factors associated with disposal of youngest child’s stools among under two years old youngsters in east Africa (unweighted *n* = 45,144)

Stool disposal variables	Null Model	Model I	Model II	Model III	*P* values
	AOR (95% CI)	AOR (95% CI)	AOR (95% CI)	AOR (95% CI)	
Maternal age					
15–24		1		1	1
25–34		1.03(0.98,1.08)		1.04 (0.99,1.09)	0.159
35–49		1.12(1.05,1.19)		**1.12 (1.05,1.19)**	**0.001**
Maternal employment					
No		1		**1**	**1**
Yes		1.32(1.26,1.38)		**1.29 (1.24,1.35)**	**0.0001**
Maternal education					
Not educated		1		1	1
Primary		1.61(1.52,1.71)		**1.62 (1.53,1.72)**	**0.0001**
Secondary/higher		1.16(1.08,1.24)		**1.22 (1.14,1.31)**	**0.0001**
Wealth index					
Poorest		1		**1**	**1**
Poorer		1.51(1.41,1.60)		**1.51 (1.42,1.61)**	**0.0001**
Middle		1.65(1.54,1.76)		**1.67 (1.56,1.78)**	**0.0001**
Richer		1.83(1.71,1.96)		**1.96 (1.82,2.11)**	**0.0001**
Richest		1.75(1.62,1.89)		**2.08 (1.91,2.27)**	**0.0001**
Mass media exposure					
No		1		1	1
Yes		1.40(1.34,1.46)		**1.37 (1.31,1.44)**	**0.0001**
ANC visit					
No		1		**1**	**1**
Yes		1.70(1.54,1.87)		**1.71 (1.55,1.88)**	**0.0001**
Place of delivery					
Home		1		**1**	**1**
Health facility		2.18(2.06,2.30)		**2.22 (2.09,2.34)**	**0.0001**
Child in months age					
< 6		1		**1**	**1**
6–11		2.12(2.00,2.24)		**2.12 (2.01,2.25)**	**0.0001**
12–23		3.09(2.93,3.26)		**3.10 (2.94,3.27)**	**0.0001**
Modern contraceptive					
No		1		1	1
Yes		1.17(1.11,1.22)		**1.17 (1.12,1.23)**	**0.0001**
Toilet facility					
Unimproved		1		**1**	**1**
Improved		1.12(1.07,1.17)		**1.12 (1.07,1.17)**	**0.0001**
Family size					
<=5		1		1	1
> 5		1.03(0.98,1.07)		1.03 (0.99,1.07)	0.196
Residence type					
Urban			1	1	1
Rural			0.89(0.84,0.94)	**0.91(0.85,0.98)**	**0.0001**
Community mass media					
High			1.33(1.22,1.44)	**1.23 (1.34,1.34)**	**0.0001**
Low			1	1	1
Community education				1	
High			1.22(1.12,1.33)	**1.33 (1.22,1.46)**	**0.0001**
Low			1	1	1
Community poverty					
High			0.78(0.71,0.84)	**0.87 (0.79,0.95)**	**0.002**
Low			1	1	1
**Random parameters and model comparison**					
Community-level variance	0.35	0.33	0.31	0.29	
ICC (%)	10.0	9.34	8.22	8.88	
MOR (%)	1.76	1.72	1.69	1.67	
PCV	Reference	5.72	11.43	17.14	
Log-likelihood (LLR)	-28,982	-26,211	-28,919	-26,137	
DIC (-2LLR)	57,964	52,422	57,838	52,274	
AIC	57,968	52,460	57,849	52,320	

### Fixed effect analysis

The odds of being exposed to safe waste disposal practices among children increased by 12% (AOR = 1.12, 95% CI, 1.05–1.19) times among women whose age is between 35 and 49 years old, as compared to women whose age is between 15 and 24 years old. Similarly, appropriate disposal was increased by 62% and 22% (AOR = 1.62, 95% CI, 1.53,1.72), and (AOR = 1.22, 95% CI, 1.14,1.31) more times among mothers who have completed their primary and secondary/higher educational attainment as compared to uneducated mothers respectively. Similar to the above, women who are members of highly educated communities have shown (AOR = 1.33, 95% CI, 1.22, 1.46) more times as compared to women from low-educated communities. Furthermore, employed women have shown 29% higher odds of practicing safe waste disposal with their children (AOR = 1.29, 95% CI: 1.24–1.35) as compared to unemployed women. Regarding household wealth index, women from poorer, middle-income, richer, and richest households have shown a higher odds ratio of safe waste disposal practices for their children as compared to poorest household wealth index mothers by the odds of 1.51 (95% CI, 1.42, 1.61), 1.67 (95% CI, 1.56, 1.78), 1.96 (95% CI, 1.82, 2.11), and 2.08 (95% CI, 1.91, 2.27), respectively. The odds of being exposed to safe waste disposal practices among children increased by 12% (AOR = 1.12, 95% CI, 1.05–1.19) times among women whose age is between 35 and 49 years old, as compared to women whose age is between 15 and 24 years old. Similarly, appropriate disposal was increased by 62% and 22% (AOR = 1.62, 95% CI, 1.53,1.72), and (AOR = 1.22, 95% CI, 1.14,1.31) more times among mothers who have completed their primary and secondary/higher educational attainment as compared to uneducated mothers, respectively. Similar to the above, women who are members of highly educated communities have shown (AOR = 1.33, 95% CI, 1.22, 1.46) more times as compared to women from low-educated communities. Furthermore, employed women have shown 29% higher likelihood of practicing safe waste disposal with their children (AOR = 1.29, 95% CI: 1.24–1.35) as compared to unemployed women. In addition to this, those women who came from high community poverty levels have shown a lower likelihood of practicing safe child feces disposal by the odds of (AOR = 0.87, 95% CI = 0.79–0.95) as compared to wealthier community women. Regarding mass media exposure status, mothers have a greater likelihood (AOR = 1.37, 95% CI: 1.31–1.44) of practicing safe waste disposal for their children as compared to their counterparts. Mass media exposure status has also shown a statistically significant association in the community-level analysis by the odds of 1.23 (95% CI, 1.34–1.34). Women who have at least one ANC visit, have utilized modern contraceptives, and have given birth to their children at health facilities have shown an odd of (AOR = 1.71, 95% CI: 1.55, 1.88), (AOR = 1.17, 95% CI: 1.12, 1.23), and (AOR = 2.22, 95% CI: 2.09, 2.34), more times to defecate their children safely, as compared to mothers who had no ANC visit, had not utilized modern contraceptives, and had given birth at home, respectively. Similarly, having an improved toilet facility has shown a positive association with appropriate waste child disposal (AOR = 1.12, 95% CI, 1.07, 1.17), more times than women who have an unimproved toilet facility. Children whose age group is 6–11 months old (AOR = 2.12, 95% CI: 2.01, 2.25), and 12–23 months old (AOR = 3.10, 95% CI: 2.94, 3.27), have shown a higher probability of appropriate waste disposal as compared to less than six-month-old children, respectively. Finally, rural women have revealed an AOR of 0.91 (95% CI, 0.85–0.98) times less than urban women (Table 3).

## Discussion

In this study, 45,144 children under the age of two who were living with their mothers in East Africa were profiled for their safe child feces disposal. Overall, 65.54% of children under two years old had their feces properly disposed. The prevalence of safe child feces disposal practices (65.54%) discovered in this study is higher than the prevalence rate of 36.9%, 59.4%, 25.5%, 56.3% and 24.5% reported from Ethiopia, Nigeria, India, Gambia and Ghana respectively [23, 42–45]. But it is lower than a study in Cambodia [32].

The following factors were identified by the study as being important major determinants of safe disposal practices as follows: residence, maternal age, education level (individual & community level), maternal employment, toilet facility, place of delivery, ANC visit, age of the child, wealth index, mass media exposure (individual and community), and community poverty.

In this study, the place of residence was related to the proper disposal of child feces. Rural residences were less likely to dispose of child feces safely. This result is in line with studies in Ethiopia [30, 42]. The rationale could be that rural residents may have less access to water, sanitation, and hygiene than residents of urban regions, which can affect how they behave in terms of hygiene as it is supported by WHO/UNICEF reports, there is a significant disparity between urban and rural people’ access to better sanitation and the practice of open defecation [46]. The variations between urban and rural areas in the use of safe child feces disposal practices may also be partly explained by the desire to adhere to particular societal norms and expectations associated with hygienic behavior [47–49].

In this study, advanced mother’s ages have been associated to safe fecal disposal practices. This finding is consistent with earlier studies. The probability that a woman has had multiple children and has developed knowledge in child rearing, including the proper disposal of children’s waste, increases with age. Additionally, the mother probably benefited from numerous health education lessons she got when visiting a medical center for child care and they may be more conscious and observant about disposing of child feces safely and are more likely to understand the causes of childhood illness [50].

The odds of safe disposal were higher as a child getting older compared to children under 6 months. This result was in line with those of a different study carried out in India, and rural Bangladesh [4, 51, 52] and Ethiopia [30]. This may be explained by the belief that young children’s stools are often seen as safe or as less harmful and nasty than those of older children [21]. Young children’s feces are smaller, smell less, and have less obvious food residue [21, 53]. The other possible reason might be after 6 months they start complementary feeding and their child feces becomes offensive which increases the likelihood of safe child feces disposal in older children as compared to younger children less than 6 month, whose child feces is not offensive making it favorable to defecate on diapers [53]. This could also be explained by a change in safe disposal behaviors that is observed as children become older; children are more likely to announce the urge of defecating and they will be collaborative [54].

According to this study, safe child feces disposal practices are more likely to be practiced when mothers have higher levels of education [32, 50, 55, 56]. These finding are consistent with earlier studies. Educated mothers are more likely to recognize the root causes of childhood illnesses and adopt hygiene practices to keep their children healthy [50]. Those educated mothers might have a chance being employed, which intern increases safe child feces disposal. Safe feces disposal was substantially correlated with the mothers’ employment status. Compared to mothers who were not employed, those who were employed had a greater probability of safely disposing of feces. This result is in line with a study in Gambia [45]. The possible reason might be that employed mothers have a source of income, can hire careers, and have easy access to sanitary supplies.

According to past studies, the practice of proper feces disposal is determined by wealth index (or household socioeconomic level) [30, 32, 57, 58]. Likewise in our study wealthier(both in the individual and community level) will have better sanitary facilities due to their higher affordability and greater drive to properly dispose of their children’s feces [59, 60]. In addition, women from wealthy families may have access to information through a variety of media sources and may therefore be aware of the significance of safe child stools disposal techniques [61, 62]. Findings showed that mothers were more likely to properly dispose of their children’s stools if they came from households with enhanced sanitation and media exposure [58]. In addition, mothers having media exposure were more likely to properly dispose of their children’s stools. This might be due to mothers who were exposed to the media may have learned vital information about child feces disposal and its effects on both the health of the child and the community as a whole. As a result, they may have better knowledge of safe child feces disposal than mothers who were not exposed, as well as a more positive attitude toward the significance of safe child disposal behavior [62].

The presence of an improved sanitation facility was significantly associated with safe child feces disposal. The result was consistent with study in Malawi and sub Saharan Africa [58, 62]. The possible explanation for this may be due to the fact that disposal of child feces in a toilet connected to a safe sanitation chain is the only safe method [63]. This contrasts with other study which stated, even individuals who have access to better sanitation facilities frequently fail to use them to dispose of child feces [42, 52, 63], and their argument to this phenomena was the presence of physical and improved sanitation infrastructure alone is not sufficient to ensure safe hygienic practices [42]. Finally, due to the fact that we employed secondary data, we were unable to add crucial variables like knowledge, perception, and other factors connected to culture and beliefs that were crucial for the outcome variable. Second, because of the nature of cross-sectional studies, it was impossible to demonstrate a temporal relationship between the independent and outcome variables. However, the study employed a sufficient sample size and standardized questionnaires. Consequently, it can be applied broadly throughout the area.

## Conclusions

East Africa has slightly poor rate of properly disposing of child feces, relative to other regions. After adjusting for confounders in both the individual and community level factors, factors like residence, maternal age, education level (both at the individual and community levels), employment of the mother, toilet facility, place of delivery, ANC visit, child’s age, wealth index, mass media exposure (both at the individual and community levels), and community poverty were significantly correlated in the final model. In order to make it more practical to dispose of child feces safely, health promotion and behavioral interventions in this area should focus on women/caregivers and their partners who lack formal education, live in rural areas, have low socioeconomic status, are unemployed, have large families, cannot access improved water, and have not been exposed to the media. Therefore, it is highly advised to strengthen and use linkages between maternal and child health care.

### Implications and the way forward

Studying safe child feces disposal in East Africa yields several implications based on the significant variables identified in the analysis.

Policy and intervention development: The findings suggest the need for targeted policies and interventions aimed at improving safe child feces disposal practices in East Africa. Efforts should be directed towards addressing factors such as toilet facility availability, maternal education, and employment opportunities to create an enabling environment for proper disposal practices.

Health promotion and education: The study highlights the importance of maternal education and awareness campaigns on safe child feces disposal. Efforts should focus on educating mothers about the health hazards associated with improper disposal and promoting hygienic practices to reduce the risk of diarrheal illnesses in children.

Infrastructure improvement: The presence of a functional and accessible toilet facility emerged as a significant factor. This finding underscores the need for investments in improving sanitation infrastructure, especially in areas with inadequate facilities. Enhancing access to clean and convenient toilets can positively impact safe feces disposal practices.

Targeting vulnerable populations: The study highlights the influence of factors such as maternal age, wealth index, and community poverty on safe feces disposal. Efforts should be directed towards supporting vulnerable populations, such as young mothers and those from economically disadvantaged backgrounds, by providing targeted interventions and resources to improve disposal practices.

Integration of health services: The associations between safe feces disposal and variables such as ANC visits and place of delivery indicate the potential for integrating health services. Health facilities can play a crucial role in promoting safe feces disposal practices by incorporating education and counseling on proper hygiene practices during antenatal and postnatal care visits.

## Data Availability

All data concerning this study are accommodated and presented in this document. The detailed data set can be freely accessible from the www.dhsprogram.comwebsite.
